# Setting priorities for land management to mitigate climate change

**DOI:** 10.1186/1750-0680-7-5

**Published:** 2012-03-16

**Authors:** Hannes Böttcher, Annette Freibauer, Yvonne Scholz, Vincent Gitz, Philippe Ciais, Martina Mund, Thomas Wutzler, Ernst-Detlef Schulze

**Affiliations:** 1 International Institute for Applied Systems Analysis, Ecosystem Services and Management Program, Schlossplatz 1, Laxenburg 2361, Austria; 2 Johann Heinrich von Thünen-Institut, Institut für Agrarrelevante Klimaforschung, Bundesallee 50, Braunschweig 38116, Germany; 3 Deutsches Zentrum für Luft- und Raumfahrt, Institut für Technische Thermodynamik, Pfaffenwaldring 38-40, Stuttgart 70569, Germany; 4 CIRED - CNRS/EHESS, 45 bis avenue de la Belle Gabrielle, Nogent s/Marne 94736, France; 5 Laboratoire des Sciences du Climat et de l'Environnement, Unité Mixte de Recherche CEA-CNRS, CE Orme des Merisiers, Gif sur Yvette, Cedex 91191, France; 6 Georg-August-Universität Göttingen, Burckhardt-Institut, Waldbau und Waldökologie der gemäßigten Zonen, Büsgenweg 1, Göttingen 37077, Germany; 7 Max-Planck-Institut für Biogeochemie, Postfach 100164, Jena 07701, Germany

**Keywords:** Carbon stock, Carbon sequestration, Carbon balance, Land management, Forestry, Agriculture, Bioenergy, Substitution, Regional modelling

## Abstract

**Background:**

No consensus has been reached how to measure the effectiveness of climate change mitigation in the land-use sector and how to prioritize land use accordingly. We used the long-term cumulative and average sectorial C stocks in biomass, soil and products, C stock changes, the substitution of fossil energy and of energy-intensive products, and net present value (NPV) as evaluation criteria for the effectiveness of a hectare of productive land to mitigate climate change and produce economic returns. We evaluated land management options using real-life data of Thuringia, a region representative for central-western European conditions, and input from life cycle assessment, with a carbon-tracking model. We focused on solid biomass use for energy production.

**Results:**

In forestry, the traditional timber production was most economically viable and most climate-friendly due to an assumed recycling rate of 80% of wood products for bioenergy. Intensification towards "pure bioenergy production" would reduce the average sectorial C stocks and the C substitution and would turn NPV negative. In the forest conservation (non-use) option, the sectorial C stocks increased by 52% against timber production, which was not compensated by foregone wood products and C substitution. Among the cropland options wheat for food with straw use for energy, whole cereals for energy, and short rotation coppice for bioenergy the latter was most climate-friendly. However, specific subsidies or incentives for perennials would be needed to favour this option.

**Conclusions:**

When using the harvested products as materials prior to energy use there is no climate argument to support intensification by switching from sawn-wood timber production towards energy-wood in forestry systems. A legal framework would be needed to ensure that harvested products are first used for raw materials prior to energy use. Only an effective recycling of biomaterials frees land for long-term sustained C sequestration by conservation. Reuse cascades avoid additional emissions from shifting production or intensification.

## Background

Land management activities are reported under the United Nations Framework Convention on Climate Change and the Kyoto Protocol as carbon stock changes in ecosystems excluding changes in the wood product pool. The effect of fossil fuel substitution is implicitly included in lower emissions from the energy sector [1]. The climate service of carbon (C) already stored in ecosystems has so far been disregarded. However, carbon stored on land can be lost by human action through harvest or removal of vegetation, the shift of forestry to shorter rotations and shorter lived products [2] and land degradation, or unwittingly through forest disturbance [3] or soil processes [4]. Ecosystems lose carbon much faster than they accumulate [5] so that the protection of the existing carbon stocks would be an alternative effective mitigation strategy in the land use sector [6,7]. Managed ecosystems usually have lower C stocks than the original natural ecosystem [6] so that every managed ecosystem carries a historical debt of C loss. Managed ecosystems, however, provide goods and services substituting energy-intensive products or fossil fuels. According to Obersteiner et al. [8] there are two principle pathways of mitigating climate change by terrestrial ecosystem management: A) improve the greenhouse gas (GHG) balance within the biosphere and B) manage for biomass production to substitute emissions from fossil fuels and sequester bio-carbon containing materials/substances outside the biosphere.

To date, no general consensus has been reached how to measure the effectiveness of climate change mitigation in the land-use sector and how to optimally distribute the various options in the managed landscape. What constitutes the most climate-friendly land use depends on 1) system boundaries, 2) time horizon, and 3) regional economic and environmental constraints.

### System boundaries

Typical system boundaries of climate change mitigation studies are: C pools in the ecosystem including live biomass and necromass, sectorial C pools including wood products, all GHGs, or indirect services by substitution of fossil energy carriers and energy intensive products. Comprehensive systems analyses addressing e.g. the project level [9], certain pathways [10], the agricultural and forestry sector as a whole [11,12], or the assessment of continental scale effects [13], reveal the true, integrated greenhouse gas performance of land management as a basis for decision making. The analysis of the full life cycle and the reuse during cascades of ecosystem products explicitly includes the substitution of energy intensive products and fossil energy sources by renewable raw materials [14-17]. Ignoring changes in the product C pool and substitution effects can significantly underestimate mitigation effects [18].

### Time horizon

Accumulation in and release of carbon from the different pools takes place at very different time scales [5] and complicates integrated assessments and appropriate comparisons. Decadal to centennial time scales reflect whether the net climate effects of land management reverse, level off or accumulate over time. Many factors determine at which point in time which mitigation options through land management are most effective. Gitz et al. [19] state that the best strategy could be to use only a minor part of the sequestration potential for slowing down the rate of growth of concentrations and the rate of abatement in the energy sector and reserving parts of the potential for the case a higher and faster decarbonization is required.

### Regional context

There is no one-fits-all strategy for optimal land management [15]. The solution will consist of a mix of land use and management systems adapted to the regional mosaic of geographical and economic constraints. Existing studies have focused on large-scale mitigation potential [20,21], economic considerations of forest rotation length with carbon taxes or subsidies [22,23], theoretical projects [24], partial aspects of forestry such as carbon removal versus timber [25,26] and bioenergy production [27,28], or agricultural land versus afforestation at global scale [29]. None of the studies covers forestry together with agricultural options at the regional level where most of the operational planning takes place. Fossil energy substitution embedded in products is an important component of effective GHG mitigation [30] but has so far been neglected in comparative studies of land use options [15,24,25,31] although studies including land use impacts in life cycle assessments exist, e.g. for agricultural bioenergy options [32].

Past management on a hectare of land has led to typical C stocks in soil, biomass and harvested products. Land management decisions start from this background. Changing the production goals from timber to energy or conservation affects all C pools and the C substitution. Land management decisions for climate change mitigation are constrained by the existing land use system and C stocks and the possible timing of management changes. Forests are characterized by high carbon stocks per hectare in soil, biomass and harvested products but relatively low productivity. Consequently, there is a risk of C loss, C sequestration potential is limited by the difference between existing and maximum achievable stable C stocks, and annual C substitution is limited by productivity. Generally, in managed landscapes, forests are located in landscape situations with productivity constraints, e.g. steep slopes or poor soils. Agricultural systems are low-carbon high-productive systems where risk of further C loss is low and annual C sequestration and C substitution potential is high.

**Figure 1:**
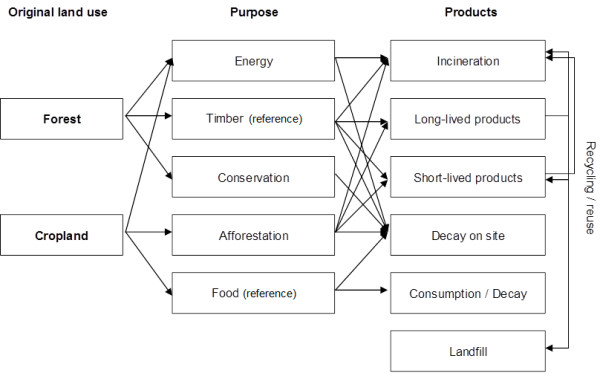
**Relations between the land management alternatives and the flow of matter through the product pools in the study**.

In this study, we analyzed a concrete complex regional situation and propose strategies to prioritize land use with regard to economic returns and climate effects using findings from life cycle assessments whilst taking existing ecosystem C stocks, productivity and costs under various system boundaries into account. We considered the traditional food and timber production as reference land use. Carbon sequestration by extensification and the switch to bioenergy by intensification and new crops were considered as alternative management systems (Figure 1) to address the following questions:

1. Exploring system boundaries: What are explicit and hidden climate effects of current and the likely best alternative management systems in agriculture and forestry?

2. Exploring time horizons: What role do efficiencies and timing of effects play?

3. Exploring constraints: How are costs and revenues affected by terrain quality, subsidies and a potential future C market?

4. Exploring opportunities: What incentives are needed to move towards an economically viable climate-protective land management?

We focused on solid biomass use for energy through combustion, because this is the most effective bioenergy option in terms of GHG abatement per unit energy and per hectare [28,33].

## Results and Discussion

The following management alternatives were compared (Figure 1):

### 

#### Forests

The two dominant forest tree species Norway spruce (*Picea abies *L.) and European beech (*Fagus sylvatica *L.) were studied in different management options:

(i) timber production as reference (*Picea *and *Fagus*: "Timber"),

(ii) shift to shorter rotations for biomass production for energy through combustion i.e. electricity production in a co-firing system (*Picea *only: "Energy") with a higher production of wood mass, and

(iii) forest conservation for C sequestration (abandonment of management, *Fagus *only: "Conservation").

*Fagus *was not considered as relevant in a pure bioenergy scenario because of its relatively slow growth in an early stage. Conversely, a sequestration scenario with *Picea *forests was not considered as feasible in the short term because *Picea *forests would require a substantial conversion to an uneven-aged, mixed forest structure to reduce the risks of major disturbances like wind fall and insect outbreaks.

#### Croplands

We studied

(i) food cereals with straw remaining on site as reference,

(ii) food cereals with straw removal for energy,

(iii) whole cereal crops for energy,

(iv) short rotation coppice of poplar for energy (clones of *Populus trichocarpa x Populus deltoides*), and

(v) afforestation with slow growing hardwood species suitable for these terrains (e.g. *Quercus robur L*.) for timber production.

### C stocks at the ecosystem and sector level

**Figure 2:**
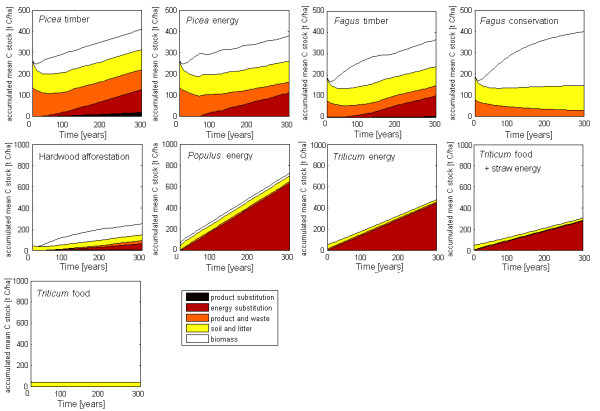
**Development of running mean carbon stocks in ecosystem and product pools plus substitution of energy and products in land use options**. Forestry options refer to medium productivity. The running mean leads to a levelling off of the typical "saw-tooth" structure of C stocks expected in forests and caused by harvest and regrowth.

Table 1 displays the C stocks in the ecosystem plus the product pool averaged over 300 years, which can be taken as equilibrium C stocks. Figure 2 shows the temporal evolution of the average C stocks in the various C pools, i.e., the C stocks in year 1 represent the start conditions and the C stocks in year 100 represent the C stocks averaged over the first 100 years of the simulations. The simulations started from the reference timber production or food cereal production at the beginning of a forest rotation or afforestation or generally, at the time of change in land management. Therefore, the initial biomass C stock was zero. The litter and soil pool and the product pool of the forestry options started at a high value due to the harvested products carried over from the year before the start of the simulation, which decayed in the first years of the simulations. For simplification, past substitution effects were ignored.

**Table 1:** C stocks, annual harvest and substitution averaged over 300 years

**Equation **3	C_ecosystem_			C_products_			ΔC_ecosystem _(300)			Substitution_produucts _(300)			Substitution_energy _(300)		
**System**	**Average C stock in ecosystem [t C ha^-1^]**			**Average C stock in products [t C ha^-1^]**			**Average annual harvest [t C ha^-1 ^yr^-1^]**			**Average annual substitution in products [t C ha^-1 ^yr^-1^]**			**Average annual substitution in energy [t C ha^-1 ^yr^-1^]**		

	**high**	**medium**	**low**	**high**	**medium**	**low**	**high**	**medium**	**low**	**high**	**medium**	**low**	**high**	**medium**	**low**

							Forestry								
*Picea*, timber	217	191	166	108	93	77	2.3	1.96	1.6	0.19	0.16	0.13	0.94	0.81	0.67
*Picea*, energy	249	217	187	59	51	42	1.9	1.62	1.3	0	0	0	0.87	0.74	0.60
*Fagus*, timber	232	216	200	50	46	43	1.9	1.76	1.6	0.04	0.04	0.04	0.88	0.82	0.76
*Fagus*,	399	372	345	28	26	24	0	0	0	0	0	0	0	0	0
conservation															
							Cropland								
Hardwood		158.5			27.9			1.09			0.04			0.48	
afforestation															
*Populus*, energy		81.1			15.0			7.57			0			4.23	
*Populus*, pulp		86.3			125.1			6.81			1.29 - 9.94			3.19	
*Triticum*, energy		21.5			12.1			6.09			0			2.96	
*Triticum*, food + energy		21.5			6.1			6.09			0			1.84	
*Triticum*, food grains		42.2			0			3.04			0			0	

The allocation of C to the various product pools, mean residence times and assumptions about reuse and recycling are given in Tables 1 and 2. High, medium and low represent different site indices of productivity for the forestry options

Since Figure 2 shows cumulatively aggregated average values for the C stocks the typical "saw-tooth" structure of C stocks in forest sites are buffered. Forestry was characterized by high C stocks in the ecosystem and product pool but small annual C stock changes due to moderate productivity and relatively long mean residence times of C in the various pools. In contrast, agricultural systems have small C pools, high productivity and high turnover rates in the biomass and product pools.

#### Forestry

The average C stocks in the *Picea *forests used for timber and energy and the *Fagus *forest used for timber were similar and ranged between 166 and 249 t C ha^-1 ^depending on the productivity level of the site (Table 1). This is in accordance with forest inventories and research plots of the region. In *Fagus *forest, conservation increased the long-term average C stocks in the ecosystems per hectare by 72% as compared to the management for timber (Table 1).

C stocks in the product pool were 48% (*Picea *timber), 23% (*Picea *energy) and 21% (*Fagus *timber) of the C pools in the forest ecosystems (Table 1). The largest fraction of the C stored in products was waste wood in landfill deposed of already prior to the start of the simulation period. The wood carbon pool in landfills is declining in Thuringia due to an introduced ban for landfilling of biomass and relatively high recycling rates of 80% [34]. The incomplete refilling of the longest lived C pool in landfills continuously decreased the product C pool in our simulations. C stocks in the product pool were highest in the *Picea *timber option due to a large fraction of harvested timber products with long mean residence times and our assumption that 20% of timber waste still ends in landfills.

The ecosystem C stocks in the *Picea *energy option were 14% higher than in *Picea *timber in the first decades and the long-term average because no thinnings were made in the energy forest. In contrast, the sectorial C stocks were 6% lower in the *Picea *energy option than in *Picea *timber because yields were lower and the wood product pool was not replenished. In *Fagus *forestry, higher C stocks in the ecosystem pools under conservation management more than compensated the C losses in the product pool. The *Fagus *conservation management resulted in a net long-term average C gain in the forestry sector by 41 to 52% above the sectorial C stocks in the timber and energy forestry.

When the C storage in the ecosystem and in the forestry sector (biomass, soil, products) was taken as system boundary the conservation of forests produced the highest climate services. This result was robust for all time horizons considered.

#### Agriculture

Agricultural lands with annual crops had five to ten times lower C stocks than forests. The removal of straw and the harvest of whole crops for energy depleted the average soil C stocks by 50% as compared to the reference of food wheat. This soil C loss is higher than the C gain in long-term agricultural experiments with extra straw over 7 to 35 years but consistent with extrapolations to a time horizon of 100 years [35]. The average ecosystem C stocks increased by 1.9 (*Populus*) to 3.8 times (afforestation) when trees were introduced (Table 1 Figure 2). Similarly, the sectorial C stocks including C stored in products were 20 to 35% lower than in the reference food crop scenario in case of straw removal but 2.3 (*Populus*) to 4.4 times (afforestation) higher. The time horizon matters in agriculture for climate services regarding C stocks. *Populus *built up the highest C ecosystem stocks during the first 33 years until the hardwood afforestation took over (not shown). However, when taking the aggregated average C stocks (Figure 2) the break-even point was only reached after 65 years. The agricultural management choices with annual crops showed only small temporal variations in the C stocks of the ecosystem and product pools due to the fast turnover of the biomass and product pools (Figure 2).

Including the C pool in products in the sectorial perspective dampened the differences in C stocks compared to the ecosystem perspective in the forestry and annual crop options. In contrast, the sectorial perspective enlarged the differences in agricultural options when trees were introduced and the mean residence time of products increased.

### Fossil C displacement

Carbon removal and storage in ecosystems tend to level off over time. So does fossil fuel substitution when more and more fossil energy is replaced and the efficiency of fossil fuel replacement diminishes. However, this depends on the development of the entire energy sector of a region and might take long. A change of the energy system over the considered horizon of 300 years is very likely, however, also unpredictable. For simplification we assume no change in the energy portfolio. A ton of carbon fossil fuel replaced cannot be reversed, that is why the climate service by substitution accumulates over time. In our case study, the substitution of coal and lignite by bioenergy achieved a substitution effectiveness above 0.8 t fossil fuel-C substituted per tonne biofuel-C harvested, substituting heating oil by biomass had an effectiveness of about 0.6 and substituting natural gas by biomass had an effectiveness of 0.4 (see Methods, Tables 4 and 5). The regional substitution effectiveness was 0.5 t fossil fuel-C substituted per tonne biofuel-C harvested for whole cereals and 0.6 in the case of wood, wood product waste and straw. The regional energy system of Thuringia has been completely rebuilt during the past 15 years and is unlikely to change much during the next decades. The substitution effectiveness is expected to decline over time because more efficient energy conversion processes will become available also for the combustion of fossil fuels. These changes in technology have not been considered here. However, the choice of solid bioenergy options and modern energy conversion technologies for heat and power plants as reference represents the most conservative estimate of the state-of-the-art with regard to regional substitution effectiveness and will therefore hold for the next decade or longer.

**Table 4:** Substitution effectiveness by fuel and conversion process combination, weighting factors reflecting the current substitutable fossil fuel mix of Thuringia and regional substitution effectiveness for Thuringia (t fossil fuel-C substituted per t of biofuel-C harvested)

Fuel	Heat plant; natural gas	Combined heat and power plant; natural gas	Combined heat and power plant; light heating oil	Heat plant. light heating oil	Power plant; hard coal	Power plant; lignite	Regional substitution effectiveness in Thuringia
*Triticum*, whole crop	0.36	0.38	0.53	0.54	0.70	0.75	**0.49**
*Populus*, short- rotation coppice	0.42	0.44	0.62	0.63	0.80	0.86	**0.57**
*Picea *, wood for energy	0.42	0.44	0.62	0.63	0.81	0.87	**0.57**
*Picea*, slash	0.44	0.45	0.64	0.65	0.83	0.89	**0.59**
*Triticum*, straw	0.45	0.47	0.66	0.67	0.86	0.92	**0.61**
**Weighting factors**	**0.27**	**0.27**	**0.11**	**0.11**	**0.07**	**0.17**	-

**Table 5:** Substitution effectiveness of product substitution in addition to energy substitution

Wood product	Substituted material	Substitution effectiveness [t fossil fuel-C substituted per t of wood-C harvested]			Reference
			
		Value used in this study	Low range	High range	
Sawn-wood:	Building construction	0.24	0.046	0.56	[54]
*Picea*	(concrete, steel, plaster)				
Sawn-wood:	Building construction	0.16	0.029	0.36	[54]
*Fagus*	(concrete, steel, plaster)				
Pulp from	Boards, pallets and pulp		0.19	1.46	[14]
*Populus*	(softwood), chemicals				

#### Forestry

The amount of energy substitution depended on the harvested biomass, the fraction of the wood and wood waste used for energy and the timing of its availability. In our Thuringian case study, the *Picea *timber option produced 10% more bioenergy than the *Picea *energy option because the growth of *Picea *was stimulated by intensive thinnings by management for timber. The amount of energy substitution by *Fagus *timber was equivalent to *Picea *timber because a large fraction of the thinning and harvest products ended in the pulp and energy segment (see Methods, Table 3). The effect of product substitution by sawn wood amounted to 20% of the energy substitution in *Picea *timber and to only 5% of the energy substitution in *Fagus *timber (Table 1). However, the product substitution effects are highly uncertain and only refer to the segment of wood products used for construction of buildings. It can be regarded as a low estimate. The combined effect of energy and product substitution by *Picea *timber exceeded the energy substitution by *Picea *energy by 32% even though 20% of the wood waste was not re-used for bioenergy.

**Table 3:** Characteristics of the land management systems

Species	System	Main product	Rotation (years)
Norway spruce (Picea abies) *SI high = 36, medium = 32, low = 28	*Picea *timber forestry	Timber (pre-commercial thinning: 0% sawn wood, 80% pulp, 20% energy; commercial thinning: 30% sawn wood, 50% pulp, 20% energy; final harvest: 80% sawn wood, 16% pulp, 4% energy; 80% of sawn wood and pulp recycled for energy)	100
	*Picea *energy forestry	100% of extracted wood for energy	60
Common beech (Fagus sylvatica) *SI high = 36, medium = 32, low = 28	*Fagus *timber forestry	Timber (pre-commercial thinning: 0% sawn wood, 50% pulp, 50% energy; commercial thinning: 10% sawn wood, 30% pulp, 60% energy; final harvest: 55% sawn wood, 15% pulp, 30% energy; 80% of sawn wood and pulp recycled for energy)	150
	*Fagus *conservation forestry	None (C removal and storage)	none
			
*Wheat (Triticum)*	*Triticum *cropland, food	Food grains, straw remains on site; grain:straw ratio = 1:1	1
	*Triticum *cropland, food + straw energy	Food grains, straw for energy grain:straw ratio = 1:1	1
	*Triticum *set-aside, energy	Whole plant for energy	1
*Poplar (Populus spec.)*	*Populus *set-aside, energy	100% of extracted wood for energy	3 × 5
*Oak (Quercus spec.) *	*Quercus *afforestation of set-aside cropland	Timber (thinnings: 0% sawn wood, 80% pulp, 20% energy; final harvest: 60% sawn wood, 40% pulp, 0% energy; 80% of sawn wood and pulp recycled for energy)	200

* Forest growth site index (average height at age 100 in m)

#### Agriculture

Per hectare, the agricultural bioenergy options except afforestation substituted 2.5 to 5.7 times more fossil C in energy than the *Picea *energy option (Table 1). *Populus *coppice had the highest average annual C substitution because of high yields and higher energy substitution effectiveness than *Triticum*.

Re-use of products along recycling cascades adds another dimension of substitution, as recycling multiplies the services of a limited biomass resource. Recycling is climate effective in most cases [14]. One additional product step can increase the annual CO_2 _emission reduction per hectare of short-rotation coppice by factor three against immediate use for energy [14]. Consequently, in our case study, if *Populus *was first used to produce pulp and then pulp waste was incinerated for bioenergy the substitution benefits could increase by factor three as compared to the immediate use for energy if high substitution effectiveness was achieved such as in the case of *Populus *wood chip incineration.

### Cumulative climate services

The cumulative climate services as expressed by the accumulated average C-stocks after 300 years ("equilibrium C-stocks"; see Methods, Equation 3) comprise the initial C stocks in ecosystem and product pools, C stock changes in these pools and the cumulative fossil C substitution by energy and products (Figure 2).

#### Forestry

In a forest rotation all management options started with the same C stock changes until the first thinning event which is necessary to produce high quality timber. This was usually at the age of 30 years (*Picea*) to 40 years (*Fagus*). The cumulative climate services in *Picea *timber were 17% higher than in *Picea *energy (Figure 2). From the time of the first thinning in *Fagus *timber onwards, the *Fagus *conservation option had higher cumulative climate services than the *Fagus *timber option throughout the simulation period. The C sequestration in the biomass of stable old-growth *Fagus *forests in the study region was higher than the C accumulation in the wood product pool and the substitution effects in the *Fagus *timber option (Figure 2). *Fagus *conservation was so effective because the mean residence times of C in the ecosystem pools were longer than those in the main *Fagus *products (see Methods, Table 3).

#### Agriculture

*Populus *coppice had by far the highest cumulative climate services throughout the simulation period, mainly due to its high substitution effects for energy or, even higher, pulp and energy (Figure 2, Table 1).

In contrast to the existing studies we have evaluated the land management alternatives by cumulative climate services averaged over decadal to centennial time scales (e.g. Figure 2) rather than by instantaneous annual climate services. This smoothed the typical "sawtooth" pattern of C stocks in forest rotations [2] and introduced a memory of the past climate performance of the land management alternatives and made the evaluation robust despite the varying rotation periods supply patterns to the product pools. Our averaged metrics are also less sensitive to assumptions about the rotation length.

**Figure 3:**
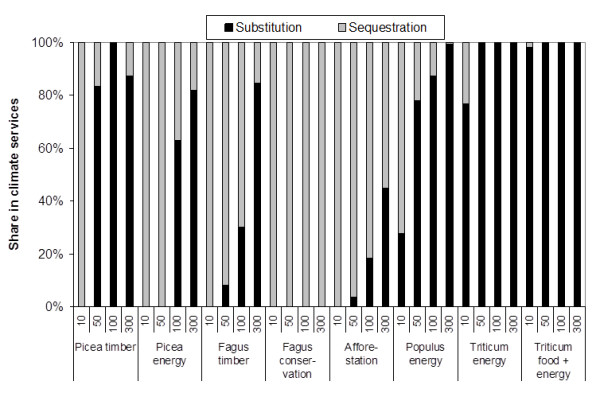
**Share of C substitution and C sequestration over time horizons of 10, 50, 100 and 300 years**.

We have tested the implications of short versus long time horizons for the produced climate services. Figure 3 shows the fractions of averaged sectorial C stock changes and C substitution services averaged over time horizons from 10 to 300 years. C sequestration dominates in *Fagus *and afforestation systems and before the first thinning or harvest in *Picea *(Figure 3). C substitution dominates in the crop options and in the timber and energy oriented forestry systems over longer time horizons. A short time horizon would favour credits for C sequestration, which is, however, a transient and largely reversible service in all production oriented systems in this study. In contrast, the C substitution service is regularly renewed so that its share in climate services increases and dominates over time (Figure 3).

### Carbon prices and net revenues

The Net Present Value (NPV) is very sensitive to the period over which net revenues are cumulated and the discount factor used [23]. In the results presented here the NPV represented the cumulative net revenue over 300 years discounted by 0.01 annually. 300 years is the least common multiple of all rotations in the analysis.

### Net revenues with a carbon price of zero

#### Forestry

**Figure 4:**
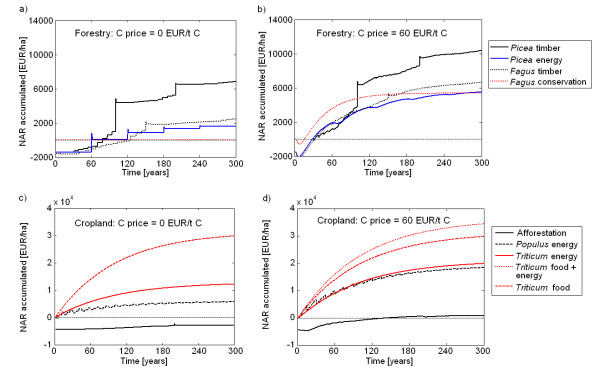
**Cumulative net annual revenue (NAR) of forestry (top: a, b) and cropland options (bottom: c, d) calculated with a discount factor of 1%, medium forest productivity, flat slope, no subsidies and a C price for removal and storage and substitution of 0 Euro per tonne C (left: a, c) and 60 Euro per tonne C (right: b, d)**. The values given at time = 300 years indicate the Net Present Value (NPV).

**Figure 5:**
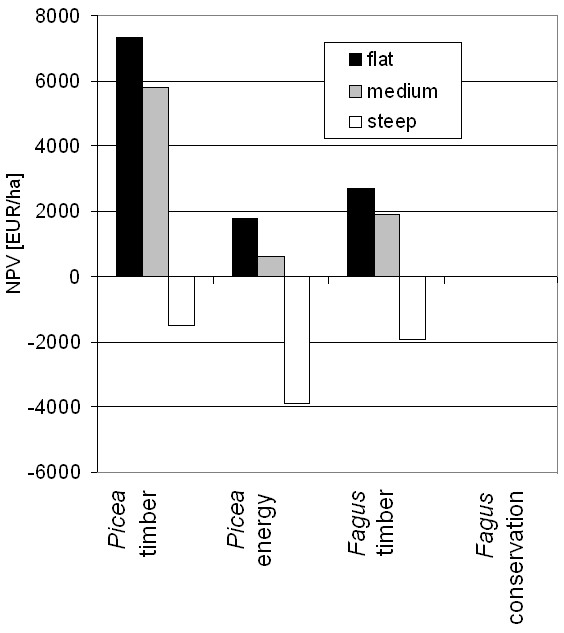
**Net Present Value (NPV) of different forestry options at medium productivity varying with slope class**.

Forestry is characterized by high production costs at the beginning and lower costs during the forest rotation, but only small revenues after thinning until the major revenue is achieved by harvest at the end of the rotation period. Therefore, at a carbon price of zero (Figure 4a) all forestry options remained deficient with respect to the net revenue throughout the first rotation. *Picea *energy remained deficient for two rotations and overall deficient on low productive sites on medium slopes. All forestry activities were deficient on steep slopes (Figure 5).

The NPV of *Fagus *conservation was zero. Timber-oriented forestry turned out as economically preferred management for *Picea *(NPV: 4,400 - 8,900 EUR ha^-1^) and *Fagus *(NPV: 1,600 - 3,100 EUR ha^-1^) on flat terrain and medium slopes (Figure 5). Lower fuel than timber prices and relatively high harvest costs turned energy forestry economically unattractive compared to timber forestry under all conditions. This situation, however, is bound to change in the near future because the demand for energy wood is increasing. At prices for energy wood above 50 EUR m^-3 ^energy forestry will be favoured over timber forestry. Presently wood for energy competes with pulp and palette production, where a cascadal use would not increase the NPV (assuming no price effects on wood products through by final energy use) of the owner but the climate effectiveness of wood production and use.

#### Agriculture

Net annual revenues (NAR) were immediately positive on cropland used for food or energy but remained negative for the afforestation (Figure 4c). The highest farm income with a NPV of 30,000 EUR ha^-1 ^was achieved by production of food wheat combined with or without straw for energy. In our simulation, food prices exceeded energy prices and the revenues from selling straw for energy happened to be equal to the costs for collecting the straw (see Methods, Table 1). The NPV of *Triticum *for energy only reached 40% and NPV of *Populus *for energy only 20% of the food wheat options.

Compared to cropland without subsidies, the area-based subsidies included (increased the NPV by about 30,500 EUR ha^-1 ^for options with food production and by 34,700 EUR ha^-1 ^in the pure bioenergy options. The food options still remained economically more attractive than the bioenergy options. Subsidies turned the NPV of afforestation slightly positive but it remained at 3,000 EUR ha^-1 ^in the range of the beech forest (Figure 4a) because an afforestation premium was granted for the first 20 years only and agricultural subsidies ceased.

### Net revenues with the same prices for C sequestration and fossil C substitution

#### Forestry

A price for C sequestration and substitution produced early revenues in forestry. This generally increased the NPV of forestry options, e.g. by more than 6,000 EUR ha^-1 ^at a C price of 60 EUR t^-1 ^C (Figure 4b). 60 EUR t^-1 ^C compensated the costs for establishment in timber and energy forestry within 30 years when the cumulative NAR reached zero. The NPV increased independently of species and products. Thus, the economically most favourable option remained *Picea *and *Fagus *timber up to a C price of 60 EUR t^-1 ^C. At higher C prices *Fagus *conservation became more profitable than *Fagus *timber.

#### Agriculture

Due to the higher productivity NPV increased more in the cropland options than in the forestry and afforestation options. Any positive C price made straw removal for energy in *Triticum *food systems attractive (Figure 4d). C prices above 45 EUR t^-1 ^C with subsidies and 60 EUR t^-1 ^C without subsidies favoured the production of energy over food and among the pure energy options, *Populus *over *Triticum*. Even at very high C prices the afforestation of cropland with slow-growing hardwood remained uncompetitive.

### Net revenues with different prices for C sequestration and fossil C substitution

**Figure 6:**
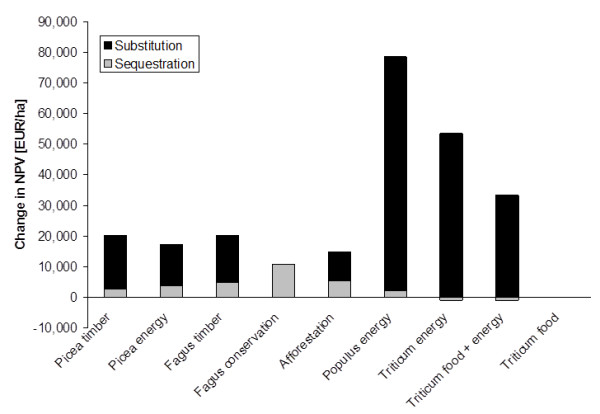
**Contribution of C sequestration and C substitution to the change in NPV at a C price of 60 EUR t-1 C**.

We can envisage market situations which include only C sequestration or C substitution or value these two climate services at different prices. The sensitivity of the management options to the C market can be tested by calculating the respective contributions C sequestration or C substitution to the change in NPV at a given C price. Table 1 demonstrates that the increase in NPV in the timber and energy options resulted almost entirely from C substitution. These land use systems are hence very sensitive to prices for C substitution paid to the producer, or similarly, to prices for bioenergy. C sequestration accounted for 50% of the increase in NPV in the afforestation and for 100% in *Fagus *conservation (Figure 6). The NPV of *Fagus *conservation was only equal or higher than of *Fagus *timber when the C price for sequestration was close to, or higher than, for C substitution. Payments for C sequestration also produced early revenues in the growth phase of forests, which were lost later at harvest and when the C stocks equilibrated over time.

### Sensitivity of results to underlying assumptions

The model simulations presented are affected by various uncertainties introduced with assumptions on productivity, substitution effects, discount factors and prices. A detailed uncertainty assessment of the model applied was presented by [36]. Effects of forest productivity levels were included by assuming three production levels (high, medium, low; see Methods for details). As can be observed from Table 1, productivity classes differed in the overall level of C services provided by the options. However, the relative ranking of options according to their climate effectiveness, however, was not changed. This is because the productivity level affects the central variable biomass and thus all other pools and climate services.

The substitution effectiveness was estimated with very detailed assumptions on the energy mix in Thuringia. The overall effectiveness of substitution depends not only on the characteristics of the reference fuel or material (e.g. fossil fuels, concrete etc.) but also on the biomass conversion pathway. For simplicity reasons we assumed only one combustion pathway. Here, many more ways of biomass conversion could be assumed we greater or smaller effectiveness. It has to be noted that substitution effectiveness will decline in the future when heat and power sector is becoming more and more effective and oil and coal are being replaced by gas and renewable energy. This effect was not included as it would require an explicit modelling of the energy sector.

The longest lived carbon pool is the product waste in landfills. The ban of organic wastes in German landfills aimed to reduce CH_4 _emissions from landfills. Alternatively, CH_4 _could be recovered and used for energy. From a mitigation point of view it could be preferable to store the product carbon in landfills rather than substituting highly efficient modern energy systems. Taking the mean substitution effectiveness of 0.5, substitution is preferable to sequestration. However, this might change in the future with more and more efficient energy production being introduced in Thuringia.

To assess the effect of uncertainty in the economic assumptions we varied the discount rate applied for the calculation of NAR and NPV as both are very sensitive to the period of accumulation and the discount factor used [23]. In forestry in Thuringia relatively low interest rates are applied, compared to agriculture and energy sector. We applied the same discount rates for all sectors to make results more comparable. The sensitivity analysis applied varying discount factors from 0.01 to 0.1. It showed that a discount factor of 0.05 was already too high to produce a profitable balance of any forestry activity. At high discount rates, management that produces early revenues or no costs (e.g. *Fagus *conservation, see Figure 4a) will be most competitive.

### Regional priorities for maximizing climate benefits

The quantitative results of system analyses as presented here are sensitive to boundary conditions, assumptions and methodological issues. Although our assumptions are based on the situation in central Germany, they give reliable relative indications of more or less effective choices [28]. In Thuringia, the highest average long-term climate services were achieved by the land use options *Picea *timber, *Fagus *conservation and *Populus *pulp and/or energy. The highest NPV was achieved by *Picea *and *Fagus *timber on flat terrain and medium slopes, no management activities in *Picea *and *Fagus *conservation, on steep slopes, and *Triticum *food with or without straw use for energy. Consequently, *Picea *forests are already managed with the highest average climate services because of the high recycling rate of wood products for energy. At the same time, forestry should develop long, effective wood recycling cascades for pulp and energy.

**Figure 7:**
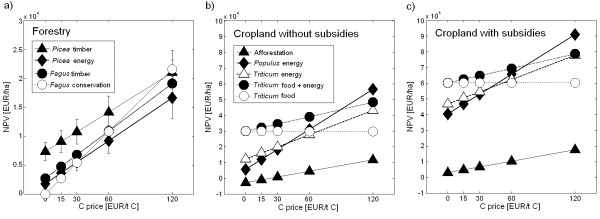
**Net Present Value (NPV) of land management options in forest (panel a) and cropland (panels b and c), with subsidies (panel b) and without subsidies (panel c) with changing price for carbon (both sequestration and substitution)**. Forestry options refer to flat slope. Error bars in a) show the variation between high and low site productivity, symbols medium productivity.

Economic constraints favor the conversion of *Picea *forests on steep slopes into stable forests for conservation, which will also produce extra climate services by C sequestration. *Fagus *conservation could be stimulated on low productive sites at marginal costs of 60 EUR t^-1 ^C for C sequestration (Figure 4 and 7). At higher productive sites, marginal mitigation costs above 100 EUR t^-1 ^C and the growing demand for wood are expected to prohibit a management change from *Fagus *timber to conservation. These rather hypothetical C prices for C sequestration and C substitution would not be competitive in the European CO_2 _emission trading system with C prices ranging from 1 to 5 EUR t^-1 ^C in 2011 http://www.pointcarbon.com. This low price is, however, not generated by a well-balanced market but the product of too many issued emission certificates and relatively low emission reduction targets. Another question is whether a reasonable incentive scheme for land owners targeting longer-term storage of carbon in landscapes should be based on market prices.

Cropland management will immediately start straw use for energy when energy prices rise or a small price for C substitution is introduced. The straw supply is, however, significantly limited by the demand for residues to maintain the soil C balance. *Populus *can only be stimulated at prices for C substitution above 80 to 90 EUR t^-1 ^C. The NPV of *Populus *and *Triticum *energy remains similar over a wide range of C prices (Figure 7). Practice will show whether a price for C substitution alone, even if it accounts for the differences in substitution effectiveness, overcomes the social barriers to grow trees or whether dedicated incentives for perennials are needed.

### Competition for productive land

In Thuringia, a strong pressure on traditional long-rotation forestry towards shorter rotations comes from growing demand for low diameter timber for modern products, such as compound wood and rising prices for energy wood. The age class distribution of Thuringian forests is unbalanced if the long rotation period is the aim [37]. *Picea *forests are dominated by young stands with moderate C stocks so that there is some flexibility with regard to the harvest age without affecting the present total regional C stocks. In contrast, old *Fagus *forests of high biological value and high C stocks are common. Shorter *Fagus *rotations would reduce total regional C stocks (e.g. also [31]). Carbon credits may be a tool to help conserve the existing large total C stocks in Thuringian forests.

Climate change mitigation adds a new demand on productive land, which is competing with the demands for food, fiber, wood and energy. Also growing bioenergy demand will compete with food and feed in the next decade [38] and increase prices for agricultural and forest commodities. Shifting land production goals from traditional goods to energy or conservation of C stocks may trigger declining C stocks, higher emissions and other environmental trade-offs elsewhere to compensate for the production losses and to satisfy human needs [39]. The high intensity of production in industrialized countries leaves little scope for further intensification or extension of productive areas without negative impacts on biodiversity, additional pollution or other negative side-effects although a separation of intensively used productive land and unproductive land for C storage and biodiversity purposes was proposed [40,41]. As a minimum requirement, climate-friendly land use would maintain the existing carbon stocks and productivity of the land.

Although the C substitution in products is uncertain, the existing case studies using life cycle assessment [14,17] agree with our finding that material use prior to energy use is more climate-friendly than dedicated bioenergy [17]. We have demonstrated for *Picea *timber *Populus *pulp that material use prior to energy use does not reduce much the biomass supply for energy if a high recycling rate is achieved. Only a clear priority for using product waste for energy together with the development of more effective product use cascades will avoid the competition between the material and energy markets. The recycling of products still offers significant easy-to-mobilize short-term potential for meeting demands for fiber, wood and energy. As demonstrated in this case study, timber and energy supply do not need to compete with each other for land. Economic incentives and legal frameworks need to be established that guarantee the highest price for land holders if they serve the long-lived, top quality segment of the market rather than directly supplying energy segments which can also be served by recycled materials. Similarly, agricultural products and residues can be used for food and fibre prior to energy use. Processing and combustion technologies are ready to deal with organic wastes so that a competition between food and energy can be reduced. More elaborate recycling cascades through various steps of wood and fiber reuse can magnify the amount of products and services derived annually from each hectare of land without increasing land use intensity.

### Generalizing the findings: a decision support system for maximizing climate benefits in managed landscapes

Fertile land is the scarcest resource in intensively managed landscapes [42]. Land use options should therefore be targeted to maximize services per unit area. Generalizing the findings of this study the most effective locations and types of mitigation measures can be identified by answering the following four questions:

1. Where is high potential for C sequestration or substitution?

• In forests: determine current, potential maximum and rotational mean carbon stocks in the forest and the current wood use portfolio.

• In agricultural land: determine regional market pressures for food and feed as indicator of possible leakage.

• If leakage risks are low, harvest levels are below sustainable cutting rates, or the current wood use portfolio has potential to increase the share of long-lived products, the fraction of available land for mitigation can be assessed.

2. Forests: C sequestration or C substitution?

• Determine the substitution effectiveness of energy wood. If the additional carbon sequestered by no-use (difference between rotational mean and maximum stable C stock in the forest) is larger than the substituted carbon in products and energy over a rotation period, C sequestration is preferable over C substitution.

• Leakage effects could mainly occur if market pressure is high and alternative forests with intensified use had higher mean rotational C stocks than the forest type chosen for C sequestration.

• Low-productive and low-access forests particularly qualify for C sequestration for economic reasons. In highly productive sites, C substitution easily exceeds C sequestration, in particular when products and energy cascades are combined.

3. Wood use for products or directly for bioenergy?

• If C substitution (in products and their re-use cascade) plus C substitution by bioenergy from re-used products exceeds C substitution by direct use for bioenergy then wood use for products is more beneficial for climate than direct use for bioenergy.

• This shows that the type of product, the re-use for other products and in particular, the fraction of wood waste that is re-used for bioenergy, determine the answer. The more wood products are re-used for bioenergy the closer comes the C substitution by bioenergy to the value of direct wood use for bioenergy.

• We showed that in the case of Thuringia, a re-use rate of 80% is sufficient to make product use more climate friendly than direct energy use of harvested wood. More elaborate re-use cascades of wood products make direct energy use inefficient in any circumstance.

4. Land use change for mitigation?

• Determine the carbon debt of land use change: How much climate benefits were generated in the previous land use system (mean C stock over rotation period, C substitution) over 20, 50, 100 years? How much climate benefits will be generated in the alternative mitigation use?

• The carbon debt of deforestation or draining high-carbon soils cannot be compensated for decades or centuries. For projects, shorter time horizons may be more relevant, which would, however, increase the carbon debt when switching from high-carbon to low-carbon systems.

## Conclusions

We used the long-term average sectorial C stocks, C stock changes, C substitution of fossil energy and energy-intensive products, and net present value as evaluation criteria for the effectiveness of a hectare of productive land to mitigate climate change and produce economic returns. We showed that these criteria should at least be averaged over one forest rotation plus the lifetime of its products so that the sectorial C gains and losses and the substitution effects are fully included.

Our results suggest that forests which can achieve stable old-growth stages and high C stocks including forests on steep slopes should be conserved for C sequestration and storage rather than harvested. When wood products are re-used for energy, there is no climate argument for switching from timber production to energy forestry. The economic conditions in Central Western Europe have already created an almost optimum climate service from forestry if energy recycling of wood products is intense. Economic incentives need to support the existing high average C stocks in long-rotation forestry and conservation, which may otherwise risk to be lost by short-term economic considerations and other land pressures. In agriculture, specific subsidies or incentives are needed to switch to perennial species for raw materials and energy, which are more effective for climate change mitigation than intensive annual crops.

In a comprehensive systems view, the production of renewable raw materials with subsequent re-use and use of residues for energy turned out as most climate-friendly and economically attractive on productive sites. Only an effective recycling frees land for long-term sustained C sequestration by conservation, or alternative non-marketable uses, beyond the present state without additional emissions from shifting production or intensification. This requires policies and economic incentives that prioritize the use for products prior to the use for energy purposes.

## Methods

### Study region

Our study is focused on Thuringia, Germany, representing a region of 16,172 km^2 ^with typical geographic and economic features of central-western Europe. Forests cover 31% of the area and are dominated by timber production from Norway spruce (*Picea abies L*., 42% of forests) and European beech (*Fagus sylvatica L*., 20% of forests). 38% of the area is under crops, mainly food cereals (62% of cropland) and an estimated fraction of 10% available for energy crops. The remaining area is used as grassland (16%), settlement and infrastructure (9%) and water bodies and other uses (5%).

Thuringian forests grow on relatively poor soils or on low mountain ranges with a wide range of environmental conditions: 28% of the forests have low, 36% medium, 36% high productivity. 26% of forests have a flat slope (< 15% inclination), 69% medium slope (15-24% inclination), and 5% a steep slope (> 24% inclination). Information about these production constraints is not publically available in a spatially explicit form. In forests, the amount of extractable products and residues is restricted by law to a level that sustains forest productivity without N fertilization. Forestry is bound by law to use indigenous tree species. Although there are exceptions (e.g. Douglas fir, *Pseudotsuga menziesii*) we did not consider planting of fast growing exotic tree species as an option at large scale. Agriculture is concentrated on the fertile lowlands. Cropland management shall maintain the existing C stocks in soils.

### Model description

We adapted the model FORMICA, a dynamic carbon-tracking model [36]. The model uses input variables derived from regional tree species-specific yield tables, forest inventory data, regional climate variables and forestry sector statistics. FORMICA calculates carbon pool trajectories under prescribed scenarios of land management. The model has a modular structure to trace C pools over time: biomass, litter, deadwood and soil, and harvested wood products. The model also accounts for the substitution of fossil fuels by wood products and bioenergy. The original forestry model [36] was extended to parameterize also agricultural options, as well as algorithms to calculate the net present value (NPV) from production costs and revenues of the various land use options.

All model input parameters and boundary conditions were based on detailed inventories and recent statistical data from forestry and agricultural operations in Thuringia, Germany. Thuringian official data sources were used for forest inventories, agricultural statistics, cost structures of farms and forest enterprises and market prices for wood, agricultural products and biofuels, and subsidies.

The model was run at annual time steps per hectare. For comparability and to account for past management, all scenarios started at the beginning of a rotation at equilibrium conditions of the reference scenarios, i.e., with equilibrium C stocks in the litter, soil and product pools and zero biomass. The model was run over 300 years, the least common multiple of the forest rotations. The model results beyond 50 years were only used to calculate average "equilibrium" C stocks and substitution effects per hectare on a uniform basis that overcomes the effects of different schedules of management and harvest activities on the studied carbon and cost variables.

### Carbon in the land use sector

The biomass module of FORMICA used static region-specific functions for species- and age-specific growth, disturbance, carbon allocation and turnover in the following biomass pools: stem wood, branches, leaves, grains, roots. The soil module YASSO [43] traced carbon through five litter and soil pools. The product module considered three product compartments for sawn-wood, pulp and bioenergy as well as partial recycling of sawn-wood and pulp for energy with regional species specific mean residence times [44,45]. We assume only recycling within a product and do not consider downgrading cascading.

The land use parts of the model were parameterized according to typical Thuringian conditions as follows: Forest management assumed regularly thinned, even-aged stands with relatively long rotations (Tables 2, 3). Growth was calculated according to regional yield tables for three site-dependent productivity levels with different yields. Three productivity levels were selected, represented by the site indices 28 (low), 32 (medium) and 36 (high). The site indices refer to the average height in meters at age 100 for spruce [46] and for beech [47].

**Table 2:** Mean residence time (MRT) of the biomass and product carbon pools in years

Species		MRT of living biomass [years]			MRT of products [years]			
	
	Stem	Branches	Leaves & grains	Roots	Saw wood	Pulp	Energy	Landfill
*Picea*	100	25	5	25	30	2	2	200
*Fagus*	150	33	1	33	25	2	2	200
*Triticum*	-	-	1	1	-	-	2	-
*Populus*	5	5	1	25	-	2	2	200
*Quercus*	200	50	1	50	40	2	2	200

MRT of stem pools is equivalent to rotation length. MRT of the other biomass pools was taken from the FORMICA model parametrized by regional studies: branches: [58], leaves and grains: [48]. For roots we assumed the same turnover as for branches due to lack of data. MRT of products was taken from [44]. The MRT of necromass is calculated by the soil model YASSO internally and cannot be displayed explicitly

The forest scenarios included a species-specific age-dependent risk of disturbance and mortality [36]. Detailed thinning and harvest schemes derived from administrative recommendations and statistics for Thuringia were implemented to assign the products from thinning and harvest removals to the fractions pulp, saw wood, and energy. Fractionation parameters depended on forest age and species. Product decay rates were adapted from a detailed regional survey [44,45]. Germany has banned by Ordinance the deposition of organic materials in landfills in 2005 so that most of the pulp and sawn-wood is disposed of by waste incineration [34]. A recycling rate for energy of 80% was used in this study, which is equivalent to the observed drop in landfilled municipal waste between 1990 and 2003 [34]. Crop yields represented the Thuringian average over the last 20 years and mean values from regional agricultural bioenergy experiments. Initial C stocks in ecosystem, product and waste pools were determined by model spin-up to species and productivity specific equilibrium levels under reference management: Timber production in *Picea *and *Fagus *forests, and *Triticum *use for food with straw remaining on site for cropland. Important management characteristics are given in Table 3. The relation between land management options and the flow of matter through the product pools and reuse loops is displayed in Figure 1.

The forest part of the model was validated separately for *Picea *and *Fagus *against a detailed carbon study based on the Thuringian forest biomass and soil inventory [37] and measured *Fagus *chronosequences [48]. Deviations between measured and modelled C stocks were within 25% for soil and within 11% for biomass without any significant bias.

### Fossil carbon displacement

To be realistic and relevant for future land management decisions and to show the possible span of climate effects we chose the bioenergy options such that they represent a modern effective energy system. The study focused on solid bioenergy used to provide electricity and heat in combustion power plants. These energy systems are several times more effective with regard to fossil carbon displacement than state-of-the-art liquid bioenergy options [28,33]. This is also the reason for ignoring the production of liquid biofuels in this study.

The CO_2 _effect of energy substitution was calculated against reference fossil fuel emission scenarios by life cycle analysis. The functional unit was the same amount and type of final energy (GJ heat, electricity or both; Table 4). Life cycle inventories were taken from [49]. We accounted for energy consumption and greenhouse gas emissions during the production, transport, provision, use and disposal of the energy carriers. The substitution effectiveness varies considerably with the type of fossil fuel and energy conversion process [50]. We therefore calculated a range of possible combinations of fossil and bioenergy carriers and conversion processes representative of the most common power plant types for heat and electricity. The effectiveness of fossil energy substitution by various solid bioenergy types, the "substitution effectiveness", was defined as "tons of avoidable fossil carbon emissions per ton of biogenic carbon harvested". The substitution effectiveness *SE *was calculated based on data from life cycle analysis [49] and additional literature [51-53], Equation 1). Data for substitution effectiveness *SE *were representative for Germany in the mid 1990s but are still valid today. Combinations of five solid bioenergy types with six fossil energy carriers in specific conversion process types were considered which are representative for the situation in Germany (Table 4).

(1) $$SE=\frac{FPE_{lc,\mathrm{ff}}+FPE_{ff}-FPE_{lc,bf}\times CER_{\mathrm{ff}}}{BC}$$

where

*SE *   Substitution effectiveness [ton of fossil energy-C substituted per ton of biomass-C harvested]

*FPEl_c,ff _*   Fossil primary energy use during the life cycle of the fossil energy carrier [GJ ha^-1 ^yr^-1^]

*FPE_ff _*   Fossil primary energy stored in the fossil energy carrier [GJ ha^-1 ^yr^-1^]

*FPE_lc,bf _*   Fossil primary energy use during the life cycle of the biogenic fuel [GJ ha- 1 yr-1]

*CER_ff _*   Carbon emission rate of fossil energy carrier [t C GJ^-1^]

*BC *   Biomass carbon harvested [t C ha^-1 ^yr^-1^]

An adequate representation of the regional fossil fuel mix substituted is important because different calorific values and carbon contents have strong influence on the regional substitution effectiveness. We assumed that fossil energy carriers were substituted proportional to their share in the regional energy balance. The regional substitution effectiveness was calculated by weighing the substitution effectiveness values, which are combination specific, according to their contribution to the Thuringian primary energy balance of stationary fossil fuel use in the year 1999 ([53]; Equation 2). The resulting regional substitution effectiveness was robust with regard to variations in the assumptions and life cycle emissions but very sensitive to the type of fossil energy carrier substituted (Table 4).

(2) $$RSE_{bf}=\sum \left(SE_{bf,\mathrm{ff}}\times w_{\mathrm{ff}}\right)$$

where

*RSE_bf _*   regional substitution effectiveness of a specific biomass type

*SE_bf,ff _*   substitution effectiveness of a specific biomass - fossil energy carrier combination and conversion process

*w_ff _*   weighting factor: relative share of the combination of fossil energy carrier and conversion process in the Thuringian fossil energy balance of 1999

For comparison, Marland and Schlamadinger [15] assume a regional substitution effectiveness of 0.6, and Dornburg & Faaij [14] a regional substitution effectiveness of 0.3 in power plants using integrated gasification combined cycle technology compared with the Western European electricity mix. In the present study the substitution effectiveness ranges from 0.49 to 0.61 depending on species and energy conversion process (Table 4).

Product substitution refers to the displacement of fossil carbon embedded in energy intensive materials such as steel and concrete by renewable energy sources such as wood. Carbon displacement factors vary in a wide range depending on the substituted good, system boundaries, allocation of energy consumption between the by-products of the life cycles, and whether the waste wood is reused for energy after demolition of the building. We took the mean value and range of the studies reviewed in [54] which exclude the reuse of waste wood because we calculated the reuse separately in our product cascade. Substitution of pulp products by poplar from short rotation coppice based on C displacement factors by [14] was additionally considered in a sensitivity analysis (Table 5). The product substitution effect occurs independently of changes in carbon stocks of the wood product pool.

### Economic analyses

The economic analyses included varying discount rates and C prices in a hypothetic C market as well as existing subsidies. The economic module of FORMICA calculated net annual revenues (NAR) with and without subsidies and their integral, the net present value (NPV) at discount rates from 0.01 to 0.1. This included production costs for all forestry and agricultural management activities (forestry: planting, fencing, thinning, harvest; agriculture: tillage, seeding, fertilizer and pesticide applications, harvest, storage if applicable). Area-related costs (e.g. planting) were distinguished from yield related costs (e.g. harvest). In forests, costs differed with type of tree removal (pre-commercial thinning, commercial thinning or harvest), productivity (3 site indices) and slope (3 classes: 0-14%, 15-24%, and > 24% inclination). Revenues comprised wood sales and sales of cereal grains for food and agricultural products for biofuels. Prices for agricultural and forest commodities were derived from regional market surveys of 2005. Table 6 shows the revenues and costs for the management options in Euro. Costs in forestry differ with slope classes. The values in Table 6 represent costs for slope class "flat" (< 15%). Costs for skidding are supposed to rise on average by 25% in slope class "medium" and 100% at "steep" slopes compared to costs listed here. These differences are due to special equipment (like winches or cable way) needed for timber extraction on steep slopes. Costs for thinning rise only in the "steep" class by 15% on average due to the increasing cost of bringing the wood to the market. Harvest costs (motor manual with chain saw) are assumed to be constant over slope classes.

**Table 6:** Revenues and costs for different management options in Euros

	Unit	*Picea*	*Picea*	*Fagus*	*Fagus*	Hardwood	*Populus*	*Triticum*	Straw	*Triticum*
		
		timber	energy	timber	conservation	afforestation	energy	energy	energy	food
Revenue land subsidies	ha^-1 ^year^-1^	0	0	0	0	0	367.1	367.1	322.1	322.1
Revenue bonus after harvest age has passed	ha^-1 ^year^-1^	0	0	0	120	300	0	0	0	0
Revenue saw wood	m^-3^	60	0	70	0	60	0	0	0	0
Revenue pulp wood	m^-3^	20	0	25	0	20	0	0	0	0
Revenue energy wood	m^-3^	30	30	30	0	30	23	66	56	0
Revenue food	(t dry matter)^-1^	0	0	0	0	0	0	0	105	105
Costs planting/establishment	ha^-1^	1450	1450	0	0	2900	322	213	213	213
Costs fencing once	ha^-1^	0	0	1600	0	1600	0	0	0	0
Costs thinning 1 (harvester)	m^-3^	11.5	0	11.5	0	11.5	17	0	0	0
Costs thinning 2 (harvester)	m^-3^	11.5	0	11.5	0	11.5	17	0	0	0
Costs harvest (motor manual)	m^-3^	14.0	14.0	14.0	0	14.0	28	56	56	56
Costs skidding	m^-3^	8.0	8.0	8.0	0	8.0	0	0	0	0

Costs differ with slope classes. The values represent costs for slope class "flat" (< 15%). Costs for skidding are supposed to rise by 25% in slope class "medium" and 100% at "steep" slopes compared to costs listed here. These differences are due to special equipment (like cable way) needed for timber extraction at steep slopes. Costs for thinning rise only in the "steep" class by 15% on average due to the need for special machines. Harvest costs (motor manual with chain saw) are assumed to be constant over slope classes

Additional region-specific subsidies for agricultural enterprises, energy crops, and afforestation were considered according to the legal situation in 2006 [55,56]. Under the European Common Agricultural Policy croplands are eligible for general area-based subsidies and extra payments for energy crops [57] of 45 EUR ha^-1 ^yr^-1 ^and of differentiated, site-, tree species- and measure-oriented payments for afforestation [55]. NPVs were first calculated without such extra subsidies and then with all subsidies included.

The changes in C stocks and the substitution effects were included in the C market, but not the initial existing C stocks. It was assumed that carbon payments were made annually. The net carbon payment (subsidies when carbon accumulates, tax when carbon is released) necessary to trigger a certain carbon objective through management change is, per definition, the mitigation cost. This was analysed by computing for each management alternative the NAR and the NPV per hectare at varying carbon prices. The marginal cost of mitigation can be derived from the difference in NPV between the management scenarios [22].

### Climate services

We determined, for time scales from a decade to centuries, the climate services of land management options in relation to different system boundaries: 1) the ecosystem perspective restricted to C stocks changes in the ecosystems, 2) the sectorial perspective including carbon storage in products, and 3) the entire systems perspective including C stock changes and greenhouse gas emissions in the life cycles of products and services and the fossil C displacement by substitution of fossil energy in power plants and of fossil energy embedded in products.

All indicators were assessed per unit of land, the scarcest resource. Indicators of climate services were calculated per hectare, annually and as cumulative values over various time horizons. Cumulative climate services were defined as

(3) $$\begin{array}{c} CS_{cum}\left(t\right)=C_{ecosystem}+C_{products}+\Delta C_{ecosystem}\left(t\right)+\Delta C_{products}\left(t\right)+\\ Substitution_{products}\left(t\right)+Substitution_{energy}\left(t\right) \end{array}$$

with

*CS_cum _(t) *   cumulative climate services at time t [t C ha^-1^]

*C_ecosystem _*   C stocks in ecosystem pools at start of simulation [t C ha^-1^]

*C_products _*   C stocks in product pools at start of simulation [t C ha^-1^]

*ΔC_ecosystem _(t) *   cumulative C stock changes in ecosystem pools until time t [t C ha^-1^]

*ΔC_products _(t) *   cumulative C stock changes in product pools until time t [t C ha^-1^]

*Substitution_products _(t) *   cumulative fossil C displacement in products until time t [t C ha^-1^]

*Substitution_energy _(t) *   cumulative fossil C displacement in energy until time t [t C ha^-1^]

The cumulative climate services hence include the C stocks initially present. This definition differs from the climate services accountable under the Kyoto Protocol which does not allow an accounting of C-stocks. However, land use decisions between C sequestration, protection of existing C stocks or use of accumulated C can only be made including existing C stocks in the pools in the calculation of cumulative climate services. In contrast, our economic calculations only evaluate the annual changes in C stocks and substitution effects.

## Abbreviations

C: Carbon; GHG: Greenhouse gas; GJ: Gigajoule; MRT: Mean residence time; NAR: Net annual revenue; NPV: Net present value

## Competing interests

The authors declare that they have no competing interests.

## Authors' contributions

HB and AF wrote most parts of the article and carried out the largest share of the analysis. They contributed to the article in equal shares. YS carried out the assessment of substitution effects for the specific situation of Thuringia. VG implemented the method for including NPV in the forestry model. MM and TW contributed data for model setup and the description of the study region. EDS and PC initialized the discussion of the topic and contributed with detailed comments and formulations to various parts of the manuscript. All authors read and approved the final manuscript.

## References

[B1] Searchinger TD Hamburg SP Melillo J Chameides W Havlik P Kammen DM Likens GE Lubowski RN Obersteiner M Oppenheimer M Philip Robertson G Schlesinger WH David Tilman G Fixing a critical climate accounting error Science 2009 326 527 528 10.1126/science.1178797 19900885

[B2] Harmon ME Ferrell WK Franklin JF Effects on Carbon Storage of Conversion of Old-Growth Forests to Young Forests Science 1990 247 699 702 10.1126/science.247.4943.699 17771887

[B3] Kurz WA Apps MJ A 70-year retrospective analysis of carbon fluxes in the Canadian forest sector Ecol Appl 1999 9 526 547 10.1890/1051-0761(1999)009[0526:AYRAOC]2.0.CO;2

[B4] Bellamy PH Loveland PJ Bradley RI Lark RM Kirk GJD Carbon losses from all soils across England and Wales 1978-2003 Nature 2005 437 245 248 10.1038/nature04038 16148931

[B5] Körner C Slow in, Rapid out-Carbon Flux Studies and Kyoto Targets Science 2003 300 1242 1243 10.1126/science.1084460 12764181

[B6] WBGU The Accounting of Biological Sinks and Sources Under the Kyoto Protocol - A Step forwards or Backwards for Global Environmental Protection? 1998 Wissenschaftlicher Beirat der Bundesregierung Globale Umweltveränderungen (WBGU), Berlin

[B7] WBGU World in Transition - Towards Sustainable Energy System 2004 London and Sterlin, VA: EARTHSCAN

[B8] Obersteiner M Böttcher H Yamagata Y Terrestrial ecosystem management for climate change mitigation Curr Opin Environ Sustain 2010 2 271 276 10.1016/j.cosust.2010.05.006

[B9] Rootzén JM Berndes G Ravindranath NH Somashekar HI Murthy IK Sudha P Ostwald M Carbon sequestration versus bioenergy: A case study from South India exploring the relative land-use efficiency of two options for climate change mitigation Biomass Bioenerg 2010 34 116 123 10.1016/j.biombioe.2009.10.008

[B10] McKechnie J Colombo S Chen J Mabee W MacLean HL Forest bioenergy or forest carbon? Assessing trade-offs in greenhouse gas mitigation with wood-based fuels Environ Sci Technol 2011 45 789 795 10.1021/es1024004 21142063

[B11] Apps MJ Kurz WA Beukema SJ Bhattia JS Carbon budget of the Canadian forest product sector Environ Sci Pol 2001 2 25 41

[B12] OECD Agriculture and Forestry: Identification of Options for Net Greenhouse Gas Reduction Annex I Expert Group on the United Nations Framework Convention on Climate Change Working Paper, 7 1997 OECD, Paris

[B13] Rose SK Ahammad H Eickhout B Fisher B Kurosawa A Rao S Riahi K van Vuuren DP Land-based mitigation in climate stabilization Energy Economics 2012 34 365 380 10.1016/j.eneco.2011.06.004

[B14] Dornburg V Faaij APC Cost and CO2-emission reduction of biomass cascading: Methodological aspects and case study of SRF poplar Clim Chang 2005 71 373 408 10.1007/s10584-005-5934-z

[B15] Marland G Schlamadinger B Forests for carbon sequestration or fossil fuel substitution - a sensitivity analysis Biomass Bioenerg 1997 13 389 397 10.1016/S0961-9534(97)00027-5

[B16] Tilman D Hill J Lehman C Carbon-Negative Biofuels from Low-Input High-Diversity Grassland Biomass Science 2006 314 1598 1600 10.1126/science.1133306 17158327

[B17] Eriksson E Gillespie AR Gustavsson L Langvall O Olsson M Sathre R Stendahl J Integrated carbon analysis of forest management practices and wood substitution Can J Forest Res 2007 37 671 681 doi:10.1139/X06-257 10.1139/X06-257

[B18] IPCC IPCC AR4 Working Group III, Mitigation of Climate Change, Chapter 9 Forestry 2007 http://www.ipcc-wg3.de/publications/assessment-reports/ar4/.files-ar4/Chapter09.pdf

[B19] Gitz V Hourcade JC Ciais P The timing of biological carbon sequestration and carbon abatement in the energy sector under optimal strategies against climate risks Energ J 2006 27 113 133

[B20] Gielen DJ de Feber MAPC Bos AJM Gerlagh T Biomass for energy or materials? A Western European systems engineering perspective Energy Policy 2001 29 4 291 302 10.1016/S0301-4215(00)00123-3

[B21] Schneider UA McCarl BA Economic potential of biomass based fuels for greenhouse gas emission mitigation Environ Resource Econ 2003 24 291 312 10.1023/A:1023632309097

[B22] Romero C Ros V Daz-Balteiro L Optimal forest rotation age when carbon captured is considered: theory and applications J Oper Res Soc 1998 49 121 131

[B23] Van Kooten GC Binkley CS Delcourt G Effect of carbon taxes and subsidies on optimal forest rotation age and supply of carbon services Am J Agr Econ 1995 77 365 374 10.2307/1243546

[B24] Garcia-Quijano JF Deckmyn G Moons E Proost S Ceulemans R Muys B An integrated decision support framework for the prediction and evaluation of efficiency, environmental impact and total social cost of domestic and international forestry projects for greenhouse gas mitigation: Description and case studies For Ecol Manage 2005 207 245 262 10.1016/j.foreco.2004.10.030

[B25] Backeus S Wikstrom P Lamas T A model for regional analysis of carbon sequestration and timber production For Ecol Manage 2005 216 28 40 10.1016/j.foreco.2005.05.059

[B26] Bateman IJ Lovett AA Estimating and valuing the carbon sequestered in softwood and hardwood trees, timber products and forest soils in Wales J Environ Manage 2000 60 301 323 10.1006/jema.2000.0388

[B27] Kirschbaum MUF To sink or burn? A discussion of the potential contributions of forests to greenhouse gas balances through storing carbon or providing biofuels Biomass Bioenerg 2003 24 297 310 10.1016/S0961-9534(02)00171-X

[B28] Sims REH Hastings A Schlamadinger B Taylor G Smith P Energy crops: current status and future prospects Glob Chang Biol 2006 12 2054 2076 doi:10.1111/j.1365-2486.2006.01163.x 10.1111/j.1365-2486.2006.01163.x

[B29] Rokityanskiy D Benitez PC Kraxner F McCallum I Obersteiner M Rametsteiner E Yamagata Y Geographically explicit global modeling of land-use change, carbon sequestration, and biomass supply Technol Forecast Soc Change 2007 74 1057 1082 10.1016/j.techfore.2006.05.022

[B30] Gustavsson L Madlener R Hoen HF Jungmeier G Karjalainen T Klöhn S Mahapatra K Pohjola J Solberg B Spelter H The role of wood material for greenhouse gas mitigation Mitig Adapt Strat Glob Chang 2006 11 1097 1127 10.1007/s11027-006-9035-8

[B31] Sohngen B Brown S The influence of conversion of forest types on carbon sequestration and other ecosystem services in the South Central United States Ecol Econ 2006 57 698 708 10.1016/j.ecolecon.2005.06.001

[B32] Brandão M Milà i Canals L Clift R Soil organic carbon changes in the cultivation of energy crops: Implications for GHG balances and soil quality for use in LCA Biomass Bioenerg 2011 35 2323 2336 10.1016/j.biombioe.2009.10.019

[B33] Farrell AE Plevin RJ Turner BT Jones AD O'Hare M Kammen DM Ethanol can contribute to energy and environmental goals Science 2006 311 506 508 10.1126/science.1121416 16439656

[B34] UBA German Greenhouse Gas Inventory 1990-2004. National Inventory Report 2006. Submission under the United Nations Framework Convention on Climate Change Federal Environment Agency, Dessau 2006 2006

[B35] Smith P Powlson DS Glendining MJ Smith JU Potential for carbon sequestration in European soils: preliminary estimates for five scenarios using results from long-term experiments Glob Chang Biol 1997 3 67 79 10.1046/j.1365-2486.1997.00055.x

[B36] Böttcher H Freibauer A Obersteiner M Schulze ED Uncertainty analysis of climate change mitigation options in the forestry sector using a generic carbon budget model Ecol Model 2008 213 45 62 10.1016/j.ecolmodel.2007.11.007

[B37] Wirth C Schulze E-D Schwalbe G Tomczyk S Weber G Weller E Dynamik der Kohlenstoffvorräte in den Wäldern Thüringens. Abschlussbericht zur 1. Phase des BMBF-Projektes "Modelluntersuchung zur Umsetzung des Kyoto-Protokolls 2003 Jena: Max Planck Institute for Biogeochemistry

[B38] OECD-FAO Agricultural Outlook 2007-2016 2007

[B39] Mayer AL Kauppi PE Angelstam PK Zhang Y Tikka PM Importing timber, exporting ecological impact Science 2005 308 359 360 10.1126/science.1109476 15831743

[B40] Huston MA Marland G Carbon management and biodiversity J Environ Manage 2003 67 77 86 10.1016/S0301-4797(02)00190-1 12659806

[B41] West TO Marland G Net carbon flux from agriculture: Carbon emissions, carbon sequestration, crop yield, and land-use change Biogeochemistry 2003 63 73 83 10.1023/A:1023394024790

[B42] Freibauer AE Mathijs E Brunori G Damianova Z Faroult E Girona i Gomis J O'Brien L Treyer S The 3rd SCAR Foresight Exercise "Sustainable food consumption and production in a resource-constrained world" European Commission - Standing Committee on Agricultural Research (SCAR) 2011

[B43] Liski J Palosuo T Peltoniemi M Sievanen R Carbon and decomposition model Yasso for forest soils Ecol Model 2005 189 168 182 10.1016/j.ecolmodel.2005.03.005

[B44] Mund MI Profft I Wutzler T Schulze E-D Weber G Weller E Vorbereitung für eine laufende Fortschreibung der Kohlenstoffvorräte in den Wäldern Thüringens. Abschlussbericht zur 2. Phase des BMBF-Projektes "Modelluntersuchungen zur Umsetzung des Kyoto-Protokolls" Förderkennzeichen 01LK9901 Vorbereitung für eine laufende Fortschreibung der Kohlenstoffvorräte in den Wäldern Thüringens. Abschlussbericht zur 2. Phase des BMBF-Projektes "Modelluntersuchungen zur Umsetzung des Kyoto-Protokolls" Förderkennzeichen 01LK9901 2006 128

[B45] Mund MI Profft I Wutzler T Schulze E-D Weber G-E Weller E Vorbereitungen für eine laufende Fortschreibung der Kohlenstoffvorräte in den Wäldern Thüringens. Abschlussbericht zur 2. Phase des BMBF-Projektes "Modelluntersuchungen zur Umsetzung des Kyoto-Protokolls" Thüringer Landesanstalt für Wald, Jagd und Fischerei, Gotha and Max-Planck-Institut für Biogeochemie, Jena. Thüringen Forst, Gotha, Jena and Gotha 2006

[B46] Wenk G Römisch Gerold D A Nicke DDR-Fichtenertragstafel Ertragstafelauszüge 1985 Fachhochschule für Forstwirtschaft in Schwarzburg

[B47] Dittmar O Knapp Lembcke G A Nicke Buchenertragstafeln Ertragstafelauszüge 1983 Fachhochschule für Forstwirtschaft in Schwarzburg

[B48] Mund M Carbon pools of European beech forests (Fagus sylvatica) under different silvicultural management Berichte des Forschungszentrums Waldökosysteme, Reihe A, Bd. 189 2004 Universität Göttingen 256

[B49] Becher S Biogene Festbrennstoffe als Substitut für fossile Brennstoffe - Energie-und Emissionsbilanzen 1998 Band 50, Universität Stuttgart

[B50] Jungmeier G Spitzer J Greenhouse gas emissions of bioenergy from agriculture compared to fossil energy for heat and electricity supply Nutr Cycl Agroecosyst 2001 30 267 273 doi:10.1023/A:1012651614688

[B51] Kaltschmitt M Kaltschmitt M, Hartmann H Grundlagen der Festbrennstoffnutzung - Begriffsdefinitionen Energie aus Biomasse - Grundlagen, Techniken und Verfahren 2001 Berlin: Springer 770

[B52] LPP L. f. p. B. Baden-Württemberg Der deutsche Wald - Holz als Rohstoff Der Bürger im Staat 1/2000 2000

[B53] TMWTA Thüringer Energiedaten 2005 Thüringer Ministerium für Wirtschaft, Arbeit und Infrastruktur http://www.thueringen.de/imperia/md/content/tmwta/publikationen/energie/energiedaten_2005.pdf

[B54] Petersen AK Solberg B Environmental and economic impacts of substitution between wood products and alternative materials: a review of micro-level analyses from Norway and Sweden Forest Pol Econ 2005 7 249 259 10.1016/S1389-9341(03)00063-7

[B55] TMLNU Förderung von Erstaufforstungen und Förderung von forstwirtschaftlichen Maßnahmen nach dem Gesetz über die Gemeinschaftsaufgabe Verbesserung der Agrarstruktur und des Küstenschutzes Thüringer Ministerium für Landwirtschaft, Naturschutz und Umwelt http://www.thueringen.de/de/tmlfun/aktuell/foerderung/eu/strukturfonds/eagfla/eagfla1/content.html

[B56] TMLNU Förderfibel 2004/2005. Ratgeber für Kommunen, Unternehmen, Verbände und Verwaltung Thüringer Ministerium für Landwirtschaft, Naturschutz und Umwelt 2004 124

[B57] BMELV Die EU-Agrarreform - Umsetzung in Deutschland 2006

[B58] Muukkonen P Lehtonen A Needle and branch biomass turnover rates of Norway spruce (Picea abies) Can J Forest Res 2004 34 2517 2527 10.1139/x04-133

